# The Neural Implementation of Surgical Expertise Within the Mirror-Neuron System: An fMRI Study

**DOI:** 10.3389/fnhum.2018.00291

**Published:** 2018-07-20

**Authors:** Ellen Kok, Anique B. De Bruin, Koos van Geel, Andreas Gegenfurtner, Ide Heyligers, Bettina Sorger

**Affiliations:** ^1^School of Health Professions Education, Maastricht University, Maastricht, Netherlands; ^2^Institut für Qualität und Weiterbildung, Technische Hochschule Deggendorf, Deggendorf, Germany; ^3^Department of Orthopedic Surgery, Orbis Medisch Centrum, Sittard, Netherlands; ^4^Department of Cognitive Neuroscience, Maastricht University, Maastricht, Netherlands

**Keywords:** fMRI, (motor) expertise, professional expertise, (orthopedic) surgery, mirror-neuron system, action observation

## Abstract

Motor expertise is an important aspect of high-level performance in professional tasks such as surgery. While recently it has been shown that brain activation as measured by functional magnetic resonance imaging (fMRI) within the mirror-neuron system (MNS) is modulated by expertise in sports and music, little is known about the neural underpinnings of professional, e.g., surgical expertise. Here, we investigated whether and (if so) how surgical expertise is implemented in the MNS in medical professionals across three levels of surgical qualification. In order to answer the more specific research question, namely, if the neural implementation of motor expertise develops in a linear or non-linear fashion, the study compares not only brain activation within the MNS related to action observation of novices and experts, but also intermediates. Ten novices (medical students), ten intermediates (residents in orthopedic surgery) and ten experts (orthopedic surgeons) watched 60 video clips (5 s each) of daily-life activities and surgical procedures each while their brain activation was measured using a 3-T fMRI scanner. An established localization procedure was followed to functionally define the MNS for each participant individually. A 2 (video type: daily-life activities, surgical procedures) × 3 (expertise level: novice, intermediate, expert) ANOVA yielded a non-significant interaction. Furthermore, separate analyses of the precentral and parietal part of the MNS also yielded non-significant interactions. However, *post hoc* comparisons showed that intermediates displayed marginally significantly lower brain activation in response to surgery-related videos within the MNS than novices. No other significant differences were found. We did not find evidence for the hypothesis that the brain-activation level in the MNS evoked by observing surgical videos reflects the level of surgical expertise in the professional task of (orthopedic) surgery. However, the results suggest a potential non-linear relationship between expertise level and MNS-activation level.

## Introduction

Surgery is a complex professional task that requires *motor expertise.* Recently, functional-neuroimaging methods have been employed to investigate the neural implementation underlying expert performance in motor tasks (for reviews, see e.g., Debarnot et al., 2014; Yang, 2015; Bilalić, 2017; Kok and de Bruin, 2017). Expertise has been generally described as ‘a process of specific adaptations to typical tasks of a domain, rather than as development of pre-existing innate abilities’ (Gruber et al., 2010): Thus, it is interesting to investigate the neural implementation of expertise. Specifically, while motor expertise received some attention in the context of sports (Calvo-Merino et al., 2005; Balser et al., 2014) and music (e.g., Fauvel et al., 2013; Brown et al., 2015), motor expertise in professional tasks (such as surgery) has so far received relatively little attention (Bilalić et al., 2015). Compared to the domains of sports and music, in which deliberate practice and expertise development typically start in early childhood, deliberate practice in surgery starts considerably later in adulthood (Bar and DeSouza, 2016). Investigating surgical skill learning can thus provide us with unique insights into the plasticity of the adult brain. Therefore, the current study focuses on the neural implementation of motor expertise in the professional task of (orthopedic) surgery. Our study employs a contrastive expertise design and incorporates three levels of expertise involving novices, intermediates, and experts. The intermediate level is of particular interest because including only experts and novices bears the risk of concluding a linear development of motor expertise. However, expertise and its neural adaptations might not develop linearly (Schmidt and Boshuizen, 1993; Gruber et al., 2010). A linear development would imply a steady increase of a particular neural implementation of expertise from the novice over the intermediate to the expert level, but other expertise effects have been shown to develop non-linearly (cf. the intermediate effect). Consequently, this study investigates differences in brain activation between experts, intermediates, and novices in the domain of orthopedic surgery.

In research on the neuroscience of motor expertise, the mirror-neuron system (MNS; Rizzolatti and Craighero, 2004) has received considerable attention (Bilalić, 2017; Kok and de Bruin, 2017). Mirror neurons were first discovered in the monkey premotor cortex (F5) using electrophysiology: they were found to fire *both* when an action was executed as well as when the monkey observed someone else performing the same action (Gallese et al., 1996; Rizzolatti and Craighero, 2004). The core network of its human counterpart includes the inferior frontal gyrus, the dorsal and ventral part of the premotor cortex, and the inferior and superior parietal lobule (Rizzolatti and Craighero, 2004; Molenberghs et al., 2012). The MNS is thought to support action observation and action understanding as well as observational learning (Rizzolatti and Craighero, 2004).

Expertise-related differences are found in regions related to the MNS (Turella et al., 2013; Yang, 2015). For example, using functional magnetic resonance imaging (fMRI) (Calvo-Merino et al., 2005) revealed that when ballet and capoeira dancers watched videos of those two dances, specific regions within their MNS, more particularly, the bilateral premotor cortex, bilateral intraparietal sulcus, right superior parietal lobe, and left posterior superior temporal sulcus, showed higher activation when observing their mastered dance as compared to the other dance. A similar design was employed by Balser et al. (2014) who asked tennis and volleyball experts to anticipate the direction of tennis and volleyball serves shown on video clips. Experts had greater activation when observing videos showing scenes of their own sport as compared to the other sport in the pre-supplementary motor area, the cerebellum, and the superior parietal lobule. Better anticipation performance correlated with stronger activation of the superior parietal lobule (being part of the MNS).

The association of increased MNS activation and increased expertise is thought to reflect the storage of motor programs in MNS-related regions (Bilalić, 2017): the MNS responds strongest to movements that are part of the individuals’ motor repertoire, suggesting that the motor repertoire of experts modulates MNS activation. However, it is of note that this conclusion is largely grounded on evidence from studies that contrasted solely experts and novices in the domains of sports and music. Very few studies included a group of intermediates in domains beyond sports and music. An interesting example that includes intermediates constitutes a pilot study by Morris et al. (2015), who investigated expertise differences in surgery in a pilot study with three undergraduate medical students (novices), three senior house officers with at least 2 years of postgraduate surgical experience (intermediates), and three surgeons with at least 5 years of postgraduate surgical experience (experts). They contrasted a finger-tapping task with a shoelace surgical knot-tying task and a task in which knot-tying had to be imagined. Experts compared to novices showed decreased blood oxygenation level-dependent (BOLD) activation in the motor cortex during knot-tying compared to finger-tapping. Furthermore, during imagining the knot-tying task significantly higher activity was revealed in the temporal parietal junction and the posterior superior temporal sulcus in experts vs. novices. Significant differences between intermediates and experts and intermediates and novices were not found, very likely due to the small sample size.

To follow up on this study, we investigated a considerably larger sample (*n* = 30) of novices, intermediates, and experts in surgery. In line with the literature on motor expertise in sports and music, we focused on motor-expertise differences in the MNS, because studies such as the one by Calvo-Merino et al. (2005) suggest an increase in MNS activation with increased expertise, but this pattern has not yet been confirmed in professional tasks such as surgery. These tasks are of particular interest because deliberate practice in these tasks starts substantially later than deliberate practice in for example dance and music. This study aims to contribute to the understanding of the development of (motor) expertise.

The present study aimed to investigate the neural implementation of motor expertise in orthopedic surgery in the MNS. In order to answer the specific research question, namely whether the neural implementation of motor expertise develops in a linear or non-linear fashion (Schmidt and Boshuizen, 1993; Gruber et al., 2010), the study compared not only brain activation within the MNS related to action observation of novices (medical students) and experts (orthopedic surgeons), but also intermediates (residents in orthopedic surgery who are currently acquiring the specific surgical proficiency). Furthermore, we looked at the correlation between MNS activation and measures of experience (in years and in estimated number of performed surgical procedures). The stimulation material used included video clips that show surgical procedures (experimental condition) and daily-life activities (control condition). We hypothesized that the brain-activation level evoked by observing surgical videos reflects the level of surgical expertise (highest for orthopedic surgeons, lowest for medical students, and intermediate for residents) while observing daily-life activities was not expected to result in activation differences across the three study groups.

## Materials and Methods

### Participants

Thirty participants (10 for each group) were included in the study. Novices were third-year medical students (two male) with an average age of 21.2 years, *SD* = 0.9. Nine novices were right-handed, one novice was left-handed. Intermediates were orthopedic-surgery residents (seven male) with an average age of 29.0 years, *SD* = 2.5. Nine intermediates were right-handed, one intermediate was left-handed. Experts were orthopedic-surgery specialists (nine male) with an average age of 46.9 years, *SD* = 10.5. Nine experts were right-handed and one expert was left-handed. The specialists worked as board-certified orthopedic surgeons for a minimum of one and a maximum of 30 years. Novices had 0 months of experience in performing orthopedic surgeries. Intermediates had an average of 3.5 years (*SD* = 2.1) of experience (minimum 0.4 years, maximum 6.04 years). Although residency takes 6 years, some intermediates had acquired additional experience during electives. Experts averaged 18.8 years (*SD* = 10.3) of surgical experience (including 5 years of residency), minimum 8.0 years, maximum 37.0 years. The estimated number of executed hip and knee surgeries was 41.9 for intermediates (*SD* = 60.73), and 1650.0 for experts (*SD* = 2165.1). The difference in number of executed surgeries between the two groups was significant, *t*_(18)_ = 2.35, *p* = 0.031. The maximum number of executed surgeries was 170 for intermediates and the minimum number of executed surgeries was 350 for experts, so the least experienced surgeon had executed more than twice the number of surgeries that the most experienced resident had executed. This study was carried out in accordance with the recommendations of the “Medical Ethics Committee of Maastricht University Medical Center” with written informed consent from all subjects. All subjects gave written informed consent in accordance with the Declaration of Helsinki. The protocol was approved by the Medical Ethics Committee of Maastricht University Medical Center.

### Experimental Design

The experiment employed a two-factorial mixed design, with the factor *expertise level* varied between participants (three levels: novices, intermediates, experts), and the factor *type of action observation* varied within participants (two levels: surgical procedures, daily-life activities). An established MNS-localization procedure was used to functionally define the MNS as the network of interest (NOI) for each participant separately. FMRI measurements encompassed eight functional runs (two MNS-localizer runs and six runs of action observation).

### Stimulation Materials and Tasks

#### MNS-Localization Procedure

We used an adapted version of an established functional-localizer task (Spunt and Lieberman, 2012) to define the MNS individually. The procedure was adapted to be bimanual because the surgical procedures and daily-life activities were also bimanual. The stimulus set consisted of five 5 s videos of a male person performing a series of button presses on two four-button boxes. Videos were filmed from a first-person perspective and were cropped to include the hands and button-boxes only. All sequences were eight presses long and simple sequences, such as pressing each button from left to right. None of the sequences required the participant to press more than one button at the same time.

**FIGURE 1:**
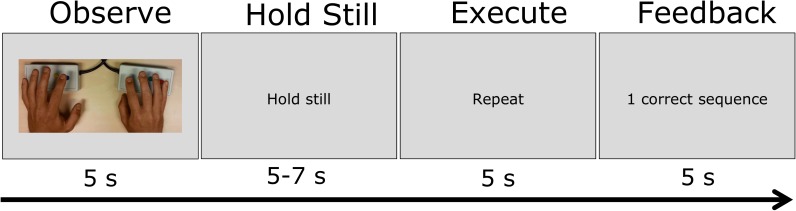
Schematic of the visual stimulation and task instruction in the MNS-localization task. In one trial, participants passively observed a hand movement sequence for 5 s, hold their hands still for 5–7 s, repeat the hand movement for 5 s and then observe the feedback for 5 s.

**Figure 1** provides an overview of a trial in the MNS-localization procedure. Participants were instructed to passively observe the sequence first (without moving their hands), then hold their hands still and only repeat the movement when the word “repeat” was presented. The sequences of button-presses were performed by participants on MRI-safe button boxes that were strapped to the participants’ legs within easy reach. After motor-task execution, participants were provided with feedback (i.e., “1 correct sequence” or “0 correct sequences”). Each trial started and ended with a 15 s baseline period (indicated by presenting a black fixation cross on a gray background). Task observation and executing were each exactly 5 s long. The time between those two was jittered 5–7 s.

The five sequences were repeated three times within a localization run in random order. The total time of each run was 9 min and 40 s.

#### Action-Observation Procedure

Three surgical procedures (two hip replacements, one knee replacement) were videotaped with a head-mounted go-pro camera (worn by an experienced right-handed orthopedic surgeon), resulting in first-person perspective video clips with a total duration of 3 h and 16 min. From this material, 60 fragments of exactly 5 s were selected, in which the surgical field (wound) and both surgeon’s hands were visible. All displayed actions were bimanual. Any other persons visible in the clips were not conducting an action other than assisting the action executed by the surgeon (e.g., holding a tool without moving).

Using the same head-mounted go-pro camera, 20 bimanual daily-life activities (listed in the Supplementary Materials) were videotaped three times when performed by a right-handed person, leading to three runs with similar but not identical video fragments. The individual 5 s video clips were assigned to six runs: one for each of the surgical procedures, and three runs of daily-life activities. The order of video-clip presentations within a run was randomized for each participant. Daily-life-activity runs and surgical-procedure runs were interleaved; half of the participants started with a daily-life-activity run and half of the participants started with a surgical-procedure run. The order of the three daily-life-activity runs and the three surgical-procedure runs within the interleaved format was counterbalanced. Between each 5 s video clip, a gray screen with a fixation cross was presented for 15 s (baseline). Participants were instructed to carefully watch the videos (focusing particularly on the two acting hands) because they would receive questions after scanning (in order to maximize attention to the visible motor action). After scanning, participants were told that they would not be questioned about the videos.

### Stimulus Presentation

Visual stimulation was generated by a personal computer (PC) using the BrainStim software^1^ and projected onto a frosted screen located at the end of the scanner bore (at the side of the participant’s head) with a liquid crystal display (LCD) projector. Participants viewed the screen via a mirror mounted to the head coil at an angle of ∼45°.

### (F)MRI Data Acquisition

Anatomical and functional brain-imaging data were obtained using a 3-T whole-body MRI scanner (Magnetom Prisma; Siemens Medical Systems, Erlangen, Germany). Participants were placed comfortably in the MRI scanner; their heads were fixated with foam padding to minimize spontaneous or task-related motion.

#### Functional Measurements

Repeated single-shot echo-planar imaging (EPI) was performed using the BOLD effect as an indirect marker of local neuronal activity (Ogawa et al., 1990). Except for the number of acquisitions (MNS-localization runs: 290 volumes; action-observation runs: 213 volumes), identical scanning parameters were used for all functional measurements (repetition time TR = 2,000 ms, echo time TE = 30 ms, flip angle FA = 77°, field of view FOV = 192 × 192 mm^2^, matrix size = 96 × 96, number of slices = 32, slice thickness = 2 mm, no gap, slice order = ascending/interleaved), runs lengths: MNS-localization run = 9 min and 50 s/action-observation run = 7 min and 16 s).

#### Anatomical Measurements

Each participant underwent a high-resolution T1-weighted anatomical scan using a three-dimensional (3D) magnetization-prepared rapid-acquisition-gradient-echo (MP-RAGE) sequence (192 slices, slice thickness = 1 mm, no gap, TR = 2,250 ms, TE = 2.21 ms, FA = 9°, FOV = 256 × 256 mm^2^, matrix size = 256 × 256, total scan time = 5 min and 5 s).

### General Procedure

After being informed about the study and having provided information about their individual surgical experience, participants were first trained in the MNS-localization task using a laptop. The procedure was identical to the procedure during fMRI scanning, except for the length of the baseline periods between two trials (which, in the training, was 2 s instead of 15 s during fMRI scanning). Verbal feedback was provided by the experimenter if participants moved their fingers during video presentation or the ‘hold-still’ period. After the practice, participants were placed in the scanner. The anatomical scan was always performed first, followed by the two MNS-localization runs and the six action-observation runs. If necessary, vision was corrected with MRI-compatible glasses. In total, the MRI session took approximately 1.5 h.

### Data Analysis

Neuroimaging data were analyzed using BrainVoyager (v20.4, BrainInnovation B.V., Maastricht, Netherlands).

#### Analysis of Anatomical MRI Data

Anatomical images were corrected for intensity inhomogeneities and spatially normalized to Montreal Neurological Institute (MNI) space.

#### Analysis of Functional MRI Data

Pre-processing of functional data included (a) slice scan-time correction, (b) 3D motion correction with intra-session alignment to the first functional volume within the MRI session, (c) temporal high-pass filtering applying a cut-off value of five cycles per run, as well as (d) spatial normalization to MNI space. Additionally, 4 mm spatial smoothing was applied to the MNS-localization data.

#### MNS Definition

For each participant, an NOI for the individual MNS was functionally defined by performing regression analysis combining the two MNS-localization runs. The general linear model (GLM) included 2 (runs) × 4 predictors according to the four conditions appearing in an MNS-localization runs (video observation, “hold-still” period, motor execution and feedback presentation). The MNS-NOIs were defined by computing a conjunction analysis contrasting 2 (runs) × 2 predictors (video observation and motor-task execution) separately against baseline. A cluster-threshold of 20 voxels was applied as well as a statistical threshold of FDR < 0.05.

Only clusters within the precentral gyrus and sulcus, inferior frontal gyrus, and parietal cortex were included in the final individual NOIs, as these are assumed to be the core regions of the MNS (Rizzolatti and Craighero, 2004). If this procedure resulted in clusters that were too extensive (e.g., the clusters in the precentral gyrus and parietal gyrus melt into each other), a more conservative Bonferroni-correction (*p* < 0.05, one-sided) was applied. This was done for six experts, two intermediates, and one novice. If a cluster encompassed <10,000 voxels, the conjunction analysis involved only two predictors (video observation and motor-task execution pooled across the two runs). This was done for one expert, two intermediates, and three novices. The choice to individually apply cluster thresholds was necessary because there were large differences in NOI-size under the same threshold. For the nine participants with very large clusters, a more conservative cluster threshold was necessary because otherwise the objective separation of the parietal and precentral part of the MNS would not have been possible: By using the FDR cluster threshold, we would have had to decide about the border by making very arbitrary and subjective decisions as to what part of the activation should be assigned to the precentral NOI, and what part should be assigned to the parietal NOI. A one-way ANOVA was used to test if any differences in final NOI-size existed between the three expertise-level groups.

#### Investigating the Effect of Expertise

An NOI-based 2 × 3 mixed random-effects ANOVA was performed involving the two predictors according to the conditions that appeared in the action-observation runs (surgical procedures and daily-life activities) and contrasting these separately against the baseline; the resulting two individual beta values per participant were extracted and further analyzed in IBM SPSS (version 22, IBM) using a 2 × 3 ANOVA as well as *post hoc* comparisons for the separate action observation types.

The same analysis was also conducted for the precentral and the parietal part of the MNS separately. This resulted in two individual beta values for the precentral part and two individual beta values for the parietal part of the MNS. Partial η^2^ was calculated as an effect size for ANOVAs. Finally, Pearson’s correlations between the beta-values and the experience of the participants (in months and in estimated number of procedures executed) were calculated.

## Results

### MNS Definition

**FIGURE 2:**
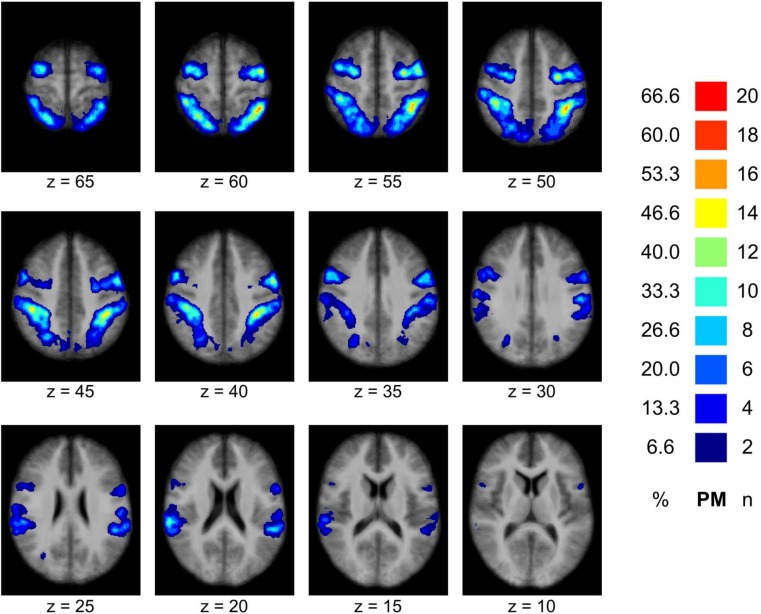
Result of the MNS definition. The figure displays a probability map of the MNS based on all individually (*n* = 30) defined MNS-NOIs demonstrating the spatial overlap of the individual MNS definitions across participants (precentral and parietal areas). The probability map is overlaid to the mean of all individual anatomical data sets. Remarks: n–number of participants showing spatial overlap of the MNS in the particular brain region; %–percentage of participants demonstrating spatial overlap in the particular brain region.

The MNS could be localized in all participants. A probability map based on the 30 individual MNS-NOIs can be found in **Figure 2**. The average NOI size (in voxels) was 32,862 (*SD* = 12,092) for novices, 36,408 (*SD* = 15,497) for intermediates and 34,624 (*SD* = 16,921) for experts. NOI size did not differ significantly between the three groups, *F*_(2,27)_ = 0.103, *p* = 0.902.

### Effect of Expertise Level

#### ANOVA and *Post hoc* Comparisons

**FIGURE 3:**
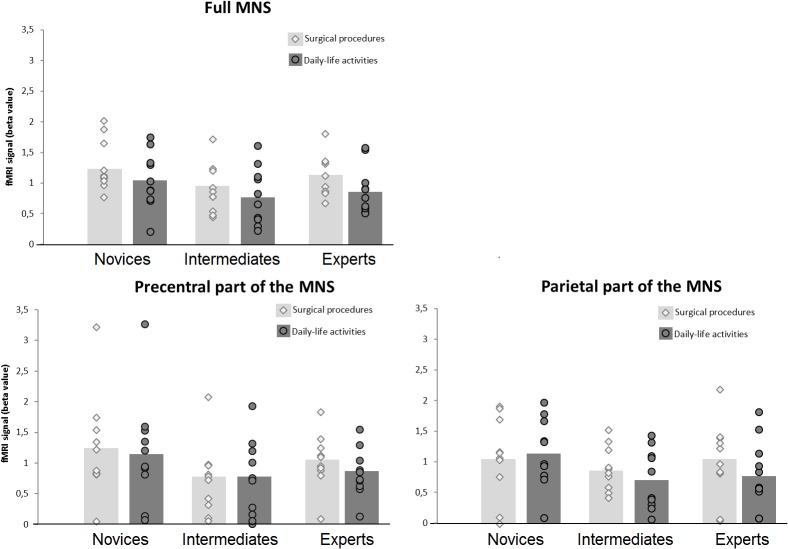
FMRI activation within the MNS. The figure displays the fMRI activation (beta value) within the MNS across the different levels of surgical expertise separately for the two types of action-observation conditions. Circles and diamonds denote individual data points. The left-upper panel displays the fMRI activation for the full MNS, the left lower panel displays the fMRI activation the precentral part of the MNS and the right lower panel displays the fMRI activation for the parietal part of the MNS.

The 2 × 3 mixed ANOVA did not reveal a significant interaction effect of *expertise level* with *type of action observation*, *F*_(2,27)_ = 0.322, *p* = 0.727, partial η^2^ = 0.023, see **Figure 3**. Also, no main effect could be ascertained for the factor *expertise level*, *F*_(2,27)_ = 1.937, *p* = 0.164, partial η^2^ = 0.125. Overall, intermediates showed the lowest beta values, *M_surgicalprocedures_ =* 0.91 (*SD* = 0.12) *M_daily-lifeactivities_* = 0.79 (*SD* = 0.15), followed by experts, *M_surgicalprocedures_ =* 1.13 (*SD* = 0.11), *M_daily-lifeactivities_* = 0.89 (*SD* = 0.12), while novices demonstrated the highest beta values, *M_surgicalprocedures_ =* 1.30 (*SD* = 0.13) *M_daily-lifeactivities_* = 1.05 (*SD* = 0.15). There was a significant main effect of *type of action observation*: *F*_(2,27)_ = 7.732, *p* = 0.010, partial η^2^ = 0.223, with videos of surgical procedures eliciting higher beta values than videos of daily-life activities.

When studying the effects of *type of action observation* more deeply, a one-way ANOVA showed no significant differences between the three expertise-level groups when observing daily-life activities, *F*_(2,27)_ = 0.823, *p* = 0.450. However, a one-way ANOVA showed marginally significant differences between the three expertise-level groups when observing surgical procedures, *F*_(2,27)_ = 2.53, *p* = 0.098. *Post hoc* comparisons between each of the three *expertise-level* groups were performed for the surgical-procedure condition against a Bonferroni-corrected *p* = 0.05/3 = 0.017. The analysis showed a marginally significant difference between novices and intermediates, *p* = 0.033. There was no significant difference between intermediates and experts, *p* = 0.220, nor between novices and experts, *p* = 0.332.

The 2 × 3 mixed ANOVA for the precentral part of the MNS did not reveal a significant interaction effect of *expertise level* with *type of action observation*, *F*_(2,27)_ = 0.432, *p* = 0.654, partial η^2^ = 0.031, see **Figure 3**. Also, no main effect could be ascertained for the factor *expertise level*, *F*_(2,27)_ = 1.585, *p* = 0.224, partial η^2^ = 0.105. There was no significant main effect of *type of action observation*: *F*_(2,27)_ = 0.967, *p* = 0.334, partial η^2^ = 0.035, with videos of surgical procedures eliciting slightly higher beta values than videos of daily-life activities.

For the parietal part of the MNS, using the 2 × 3 mixed ANOVA, no significant interaction between *expertise level* and *type of action observation* was found, *F*_(2,27)_ = 1.259, *p* = 0.300, partial η^2^ = 0.085, see **Figure 3**. Also, no main effect was ascertained for the factor *expertise level*, *F*_(2,27)_ = 0.943, *p* = 0.402, partial η^2^ = 0.065. There was no significant main effect of *type of action observation*: *F*_(2,27)_ = 2.133, *p* = 0.156, partial η^2^ = 0.073.

#### Correlations

We correlated the obtained level of the fMRI signal (beta values) with the duration of surgical experience (months of experience and executed procedures). The beta value for the surgical procedures condition did not correlate significantly with the number of months of experience (*r* = 0.072, *p* = 0.707), nor with the estimated number of executed procedures (*r* = 0.257, *p* = 0.171).

## Discussion

The present study aimed to investigate the neural implementation of motor expertise within the MNS in (orthopedic) surgery. To address this question and as an extension to the existing body of research (Bilalić, 2017; Kok and de Bruin, 2017), we included a group of intermediates, next to novices and experts, who are still acquiring surgical proficiency (orthopedic residents). This enabled us to specifically examine whether surgical motor expertise develops in a linear or non-linear fashion (Schmidt and Boshuizen, 1993; Gruber et al., 2010). A linear development would imply a steady increase of a particular neural implementation of expertise from the novice over the intermediate to the expert level. This would reflect the linear build-up of a surgery-related motor repertoire.

In our current study, we focus on the MNS as previous research (Calvo-Merino et al., 2005) indicated a crucial involvement of this brain network in the context of motor expertise. We used an adapted version of an established localization procedure (Spunt and Lieberman, 2012) to functionally define the individual MNS for each participant. We hypothesized that the brain-activation level evoked by observing videos of surgical procedures reflects the level of surgical expertise in a linear fashion (highest for orthopedic surgeons, lowest for medical students, and intermediate for residents) while observing daily-life activities was not expected to result in activation differences across the three groups.

The 2 × 3 mixed ANOVA showed neither a significant main effect of *expertise level* nor a significant interaction effect, but it showed a significant main effect for the factor *type of action observation* (surgical procedures vs. daily-life activities). Separate ANOVAs for the daily-life activities condition and the surgical-procedures condition showed no significant effects of *expertise level* in the daily-life activities condition, as expected, but a marginally significant main effect of expertise level in the surgical-procedures condition. Bonferroni-corrected *post hoc* comparisons within the surgical-procedure condition showed that intermediates displayed marginally significantly lower levels of MNS activation than novices did. Descriptive analyses showed higher levels of MNS activation in experts vs. intermediates, but this difference did not reach significance. Because the three groups showed comparable levels of MNS activation when watching daily-life activities, this suggests that the neural implementation of motor expertise in surgical procedures might potentially develop non-linearly (Schmidt and Boshuizen, 1993; Gruber et al., 2010). Separate 2 × 3 mixed ANOVAs for the precentral and the parietal part of the MNS did not show significant main effects for *expertise level, type of action observation* or an interaction between those two factors. Thus, we did not find evidence for the hypothesis that the brain-activation level evoked by observing surgical videos reflects the level of surgical expertise in the professional task of (orthopedic) surgery.

Our results are surprising in light of the findings by Calvo-Merino et al. (2005) and others (Yang, 2015). Previous research found the MNS to be very specific in its activation, responding more strongly to movements that were part of participants’ motor repertoire than to those that were kinematically comparable, but not part of the motor repertoire. Our study has a set-up that was very similar to that of Calvo-Merino and colleagues, using a video-observation task, too, as well as involving a similar number of participants in each group. In contrast to their findings, however, we did not find differences between experts and novices when watching videos of surgical procedures. We did find the intermediates to show somewhat lower activation levels compared to novices, whereas previous studies had found increased higher activation levels in more experienced participants. This suggests a different neural representation of motor expertise in professional tasks.

An advantage of the present study in comparison to previous studies investigating the neural implementation of motor expertise is that we included a group of intermediates. As suggested by Gruber et al. (2010) as well as Schmidt and Boshuizen (1993), only including (at least) three levels of expertise in the study design allows an examination of non-linearity of motor-expertise development. Our results suggest a potential non-linear neural implementation of surgical-motor expertise within the MNS. The potential non-linearity of motor expertise can be explained in the context of studies by Liew et al. (2013) and Vogt et al. (2007), who found that the MNS also responds to novel movements. This response tended to decrease with extended viewing. In order to better understand how the brain response within the MNS is modulated by developing motor expertise, it is important that further research makes extended efforts in sampling participants from multiple levels of expertise and not only experts and novices. Another advantage of the present study was the use of a surgical task. In addition to the larger body of evidence in domains of sports and music (Calvo-Merino et al., 2005; Brown et al., 2015), in which the development of expertise tends to start at a very early age, the development of motor expertise in surgery starts considerably later, typically after graduating from higher education. Thus, the use of surgical stimuli affords unique insights into the developing plasticity of the motor system in the adult brain.

In the present study, the response of the MNS to the surgical-procedure videos was larger than for the daily-life videos. This was a consistent pattern of findings in all three groups, although this difference did not reach significance in the separate analyses of the precentral and parietal part of the MNS. The two types of videos were similar in that they were both filmed using a go-pro camera (first-person perspective); in addition, all movements were bimanual, and in all movements tools (e.g., a knife) were manipulated by a right-handed person. What explains the increased MNS activation related to the surgical procedures? First, it is possible that, in general, surgical procedures are more complex to execute than daily-life activities. Second, it is also possible that all participants (even the surgeons) have more experience with daily-life tasks (such as pouring in drinks) than with surgical tasks and learned these tasks at a younger age. Third, participants could have paid more attention to the surgical procedures than to the daily-life tasks, for example because they were more interesting to them. Finally, motivation could also explain this effect (c.f., Cosic et al., 2012), as participants may have identified themselves more with the surgical-procedure videos than with the daily-life-activity videos.

It could be argued that the novices, intermediates, and experts in our sample also have different levels of experience with *observing* the surgical procedures, which could explain the results. For example, our intermediate group consists of residents, who spend a lot of time observing the surgical procedures. Calvo-Merino et al. (2006) investigated a similar explanation in an elegant study in dance. Both female and male dancers observed gender-common and gender-specific dance movements. Dancers have extensive experience observing the gender-specific dance movements of the other gender, but only possess a motor representation for gender-common movements and the gender-specific dance movements of their own gender. Greater MNS activation was found for those movements that were part of participants’ own motor repertoire as compared to opposite-gender moves (that they normally only observe). Thus, this suggests that our results reflect the motor repertoire of our participants, and not their observational experience.

An important difference between our study and the studies by Calvo-Merino et al. (2005, 2006) is that our study involved mostly hand-eye coordination whereas their studies involved whole-body movements. This might results in smaller parts of the MNS being affected by the development of motor expertise, and thus in less noticeable differences in MNS activation. Alternatively, the findings of Calvo-Merino et al. (2005, 2006) might not apply to hand-eye coordination and mostly to coarser whole-body movements. Since our MNS localizer was also a fine-grained, bi-manual task, it was likely to have activated those regions of the brain that would be involved in surgery-related hand-eye coordination. Further research involving fine-grained movements in tasks that people learn at a younger age (e.g., violin or guitar playing or crafting) could shed light on the question whether the Calvo-Merino’s findings are limited to whole-body movements, or whether they are limited to expertise that is developed from a young age.

A limitation of this study is that we investigated motor expertise without allowing participants to actually perform the acquired professional motor skills. Movements in the scanner would have had deleterious effects on data quality of this study. This limitation is affecting research on motor expertise in general (Mann et al., 2013), and different solutions have been suggested and applied for this problem (Kok and de Bruin, 2017). Some studies have investigated surgery with tasks that did require participants to make domain-related movements: For example, Bahrami et al. (2011) have built an fMRI-compatible laparoscopic surgery trainer to allow non-expert participants to train surgical movements while being scanned (Bahrami et al., 2014). Morris et al. (2015) had participants tie knots on a jig. In both cases, however, only relatively simple movements could be performed and they were not executed in the complexity of a surgical procedure, where movements rely on visual cues. While our solution taps into higher-level processes related to motor expertise (in particular related to visual information), the disadvantage of our approach is that it does not require participants to actually plan and execute movements. Given that each solution taps into a different aspect of what constitutes expertise, it is critical that researchers take different approaches when investigating the neuroscience of motor expertise, so we can understand under which conditions the findings converge and diverge (de Bruin, 2016).

## Conclusion

This study is among the first to investigate the neural implementation of motor expertise in a professional, here surgical task and the first to include a considerable sample of participants with an intermediate level of expertise (a pilot-study of Morris et al., 2015 only included three intermediate participants). A particular focus was put on the MNS as it was previously found to selectively respond to movements that are part of an expert’s motor repertoire. Our findings suggest that intermediates showed the lowest activation in response to surgery-related videos, with novices and experts showing non-significant differences. In contrast to work by Calvo-Merino et al. (2005), we did not find an increase in MNS activation with increased expertise. However, our results suggest a potential non-linear pattern (Schmidt and Boshuizen, 1993; Gruber et al., 2010) in MNS-activation with increasing expertise in a domain-specific action-observation task.

## Author Contributions

EK, ADB, IH, and BS were involved in designing the work. EK, KvG, AG, and BS were involved in data acquisition. EK and BS were involved in analysis and interpretation of the data. EK drafted the work. BS, IH, ADB, AG, and KvG critically revised the manuscript. All authors approved the final version of the manuscript.

## Conflict of Interest Statement

The authors declare that the research was conducted in the absence of any commercial or financial relationships that could be construed as a potential conflict of interest.

## Footnotes

1 https://github.com/svengijsen/BrainStim
