# Supplementary material for: The Neural Implementation of Surgical Expertise Within the Mirror-Neuron System: An fMRI Study
Source: Front Hum Neurosci. 2018 Jul 20;12:291. doi: 10.3389/fnhum.2018.00291 (PMC6062624; doi:10.3389/fnhum.2018.00291)
Supplement: Supplementary file 1 [file Table_1.DOCX]

Supplementary Material

**The Neural Implementation of Surgical Expertise within the Mirror-Neuron System: An fMRI Study**

**Ellen M. Kok*, Anique B. H. de Bruin, Koos van Geel, Andreas Gegenfurtner, Ide C. Heyligers, Bettina Sorger**

*** Correspondence:** Ellen Kok: e.m.kok@uu.nl

# Supplementary Tables

**Table I: Overview of the daily-life activity videos**

| 1. Opening a book |
| --- |
| 1. Shuffling a deck of cards |
| 1. Putting a DVD in its cover |
| 1. Pouring tea |
| 1. Lighting a candle |
| 1. Pouring a drink from a carton |
| 1. Typing |
| 1. Opening a jar |
| 1. Spooning sugar in a cup of tea |
| 1. Stapling papers |
| 1. Washing hands |
| 1. Putting toothpaste on a toothbrush |
| 1. Drying glasses |
| 1. Doing the dishes |
| 1. Typing on a calculator |
| 1. Making holes in a paper |
| 1. Sharpening a pencil |
| 1. Putting scotch tape on a present |
| 1. Highlighting words in a text |
| 1. Using a measuring tape |
